# The heart team: the multidisciplinary approach to coronary artery disease

**DOI:** 10.20517/2574-1209.2023.122

**Published:** 2024-01-24

**Authors:** Ramon A. Riojas, Jennifer S. Lawton, Thomas S. Metkus

**Affiliations:** 1 Division of Cardiac Surgery, Department of Surgery, Johns Hopkins University School of Medicine, Baltimore, MD 21287, USA.; 2 Malcolm Grow Medical Clinics and Surgery Center, Joint Base-Andrews, MD 20762, USA.; 3 Divison of Cardiology, Department of Medicine, Johns Hopkins University School of Medicine, Baltimore, MD 21287, USA.

**Keywords:** Multidisciplinary team, heart team, complex coronary artery disease, PCI, CABG

## Abstract

The recommendation to employ a heart team to guide revascularization has persisted for over a decade. Despite evidence for improved adherence to guidelines, widespread adoption of the heart team approach has been limited. This review delves into the history of the guidelines endorsing the use of a heart team and the supporting data. Additionally, it outlines some attributes of a successful heart team, and how the heart team has been run at several large academic centers. Finally, it reviews some of the barriers to a heart team and future considerations.

## INTRODUCTION

Many medical fields have adopted team-based approaches to decision making due to rapidly evolving and complex treatment algorithms. Guidelines are produced by major societies, and new algorithms are published almost annually^[1]^. Risk score calculators are generated to help with decision making. As such, navigating the decision-making process by a single physician or healthcare provider is ever challenging. In the field of cardiovascular disease, many institutions have developed multidisciplinary teams of physicians and providers to help with the decision-making process^[2–5]^. For example, cardiologists and surgeons meet with a variety of other health providers to determine whether patients with end-stage heart failure are candidates for ventricular assist devices or cardiac transplantation. Multidisciplinary teams are essential for determining whether patients undergo surgical or transcatheter aortic valve and mitral valve replacement^[6]^. Some hospitals have “shock” teams to help determine how to manage patients in acute cardiogenic shock^[7]^. The pulmonary embolism response teams (PERT) decide how to manage patients with severely symptomatic, large pulmonary emboli^[8]^. Thus, the role of a single provider making a life-changing, complex decision has evolved into multidisciplinary teams contributing information and analyzing data from different perspectives to determine the optimal treatment pathway.

In coronary artery disease, there are primarily three treatment pathways: medical management, percutaneous coronary intervention by an interventional cardiologist, or coronary artery bypass grafting by a cardiac surgeon. Since most patients are seen by a primary cardiologist, this person has a large influence on the referral pattern and decision making for their patients. Next, an interventional cardiologist may perform a diagnostic coronary angiogram, and if there is significant coronary artery disease, he or she may decide to perform an intervention without further discussion, also known as *ad hoc* PCI^[9–14]^. When a patient is referred to a cardiac surgeon, the surgeon will review the coronary angiogram to determine possible targets for bypass grafting and, at the time of surgery, will make a final decision on whether these targets are suitable^[15,16]^. All of these are examples of individual decision making in coronary artery disease. There are many instances where cardiologists and surgeons work in a collaborative atmosphere and may have impromptu discussions on how best to manage certain patients^[9]^. Many hospitals have now developed a multidisciplinary heart team that presents, reviews, discusses, and collectively decides on the best evaluation and treatment modality based on the data available^[2,17–19]^. Such heart teams utilize combined decision making to determine the best treatment strategy for complex patients with coronary artery disease. This narrative review will discuss data supporting the use of a heart team, how to implement a heart team, some barriers to the heart team, and future considerations. A topic-based search of the Pubmed database was performed using keywords such as “multidisciplinary team, heart team, complex coronary artery disease, PCI, and CABG”.

## A BRIEF HISTORY OF THE HEART TEAM

There is large variability in physician-made decisions for the management of CAD, raising concerns about inappropriate revascularization^[2]^. Some reports have shown the percentage of *ad hoc* PCI to be over 70% of cases^[12,13,20]^. Many of these cases were in complex lesions including 2- or 3-vessel CAD, left main coronary artery disease, and chronic total occlusions, in which some studies have shown better outcomes with CABG. Thus, the major question is whether the optimal treatment modality was chosen^[10]^. One study showed that patients undergoing coronary angiography in hospitals with a higher PCI:CABG ratio had increased rates of major adverse cardiac and cerebrovascular events (MACCE) and repeat revascularization 29. Additionally, there was an increased hazard rate of MACCE, death, or MI if the patient had the index angiogram at a hospital without CABG capability *versus* at a hospital with CABG capability. This raises the question of whether there was an effective multidisciplinary discussion of the management of CAD in hospitals without CABG capability. In contrast, multiple observational studies have demonstrated favorable and reproducible outcomes when a Heart team was used in the evaluation of patients with coronary artery disease. The earliest data were derived from studies comparing percutaneous transluminal coronary angioplasty (PTCA) to CABG for revascularization. For example, the EAST^[21]^, GABI^[22]^, and BARI^[23]^ trials were randomized controlled trials designed to compare CABG and PTCA. Patients had to meet criteria for both PTCA and CABG and were evaluated by both cardiologists and surgeons. The BARI trial had very specific criteria for the experience level and recent outcomes of the interventional cardiologists and surgeons^[24]^. These trials included nested registries along with randomized cohorts to evaluate physician or patient treatment preferences *versus* patients in whom clinical equipoise was assumed^[2]^. The registry patients demonstrated increased 3-year survival in the EAST registry and increased 7-year survival in the BARI trial, suggesting that selection of treatment after discussion with a cardiologist, cardiac surgeon, and patient yields better outcomes compared to randomization.

## CLINICAL EVIDENCE SUPPORTING A HEART TEAM

Since then, data has accumulated regarding the efficacy of a heart team. In 2012, Chu *et al*. at the University of Pittsburgh Medical Center formed a multidisciplinary heart team to discuss patients with complex coronary artery disease^[3,18]^. In this pilot study, 180 patients were included, and 36% underwent PCI, 48% CABG only, 2% hybrid approach, and 14% medical therapy alone. The 30-day mortality was 8% for the PCI group, 1% for the CABG-only group, and 12% for the medical management-only group^[3]^. This was one of the first pilot prospective cohort studies, and the authors concluded that implementation of a Heart team is feasible, safe, and efficacious^[3]^. Several other observational studies of complex coronary artery disease also showed better adherence to guideline recommendations and appropriateness of intervention when a heart team was utilized^[4,19]^.

Even after the initial published recommendations for a heart team in 2010^[25]^, there still was a lack of randomized data regarding the benefits of a heart team discussion or involvement in patient care. Yamasaki *et al*., retrospectively reviewed their clinical outcomes 2 years before and 2 years after implementation of a heart team^[26]^. Notably, the PCI:CABG ratio decreased after the implementation of the heart team. A multivariable analysis demonstrated that the number of MACCE was reduced with the heart team approach. Further, after propensity score matching, the heart team approach was independently associated with reduced MACCE.

A retrospective review of the Critical Care Cardiology Trials Network registry evaluated the use of Shock teams in cardiac intensive care units between 2019 and 2021. Patients in cardiogenic shock treated at hospitals with shock teams had shorter median ICU length of stay and less mechanical ventilation. In this study, the presence of a shock team was independently associated with lower ICU mortality (adjusted OR 0.72; 95%CI: 0.55–0.94, *P* = 0.016)^[27]^.

Although the above data suggest improved long-term outcomes and improved adherence to current guidelines when a heart team is utilized, there is no randomized controlled trial evaluating the outcomes of a heart team *versus* no heart team discussion of patients with CAD.

## GUIDELINES SUPPORTING THE USE OF A HEART TEAM

Whereas there may be no perfect method for making recommendations for the treatment of coronary artery disease, the medical community should work towards creating a bias-free decision-making process. The “Heart team” is not a novel concept, and in fact, it has been trialed at many major institutions^[5,17,18,28,29]^. The concept of the heart team approach was developed in the context of randomized controlled trials comparing PCI with CABG. In these trials, the Heart team would ensure that patients were equally suitable for randomization to either PCI or CABG^[30,31]^. Following these studies, it became evident that the use of a collaborative heart team could help in decision making between providers and patients^[2]^. Additionally, there have been previous reports about the inappropriate use of PCI or CABG with marked differences in European countries^[10]^. Thus, the 2010 ECS/EACTS Guidelines for myocardial revascularization made a Class 1 recommendation based on Level C evidence for the use of a Heart team to guide institutional protocols and discuss complex patients on an individual basis^[25]^. These recommendations have been promoted in the 2012 ACCF/SCAI/STS/AATS/AHA/ASNC/HFSA/SCCT^[32]^, and 2014 and 2018 ECS/EACTS guidelines^[11,33,34]^. The most recent guidelines to recommend the use of a multidisciplinary Heart team to discuss revascularization is the 2021 ACC/AHA/SCAI Guideline for Coronary Artery Revascularization^[1,35]^. In the 2021 report, the recommendation for a Heart team was given a Class 1 recommendation based on Level B-NR evidence, and recommends representatives from interventional cardiology, cardiac surgery, and clinical cardiology, as well as other specialists that may be involved in the care of the patient. The Heart team must continue to stand on the pillars of commitment to excellence in patient care, mutual respect amongst medical providers, and fair and equitable decision making for all patients. In addition to discussing the complex coronary artery disease, the team should consider comorbid conditions that may affect the revascularization strategy, and other clinical or social factors that may impact the desired outcome [Table 1]^[35]^. Ideally, this collaborative approach will provide patients with evidence-based and unbiased treatment choices. Furthermore, these options should involve a shared decision-making process with the patients and align with their personal values and preferences. The patient-centric model of including the patients and their support system in the decision-making process is also a Class I recommendation^[1]^.

Soon after the 2014 guideline recommendations for a heart team, a working group on behalf of the British Cardiovascular Society (BCS), Society for Cardiothoracic Surgery in Great Britain and Ireland (SCTS), and British Cardiovascular Intervention Society (BCIS) developed guidance on how their heart teams should function. In addition to a designated chairperson and attendance by cardiologists and surgeons, the BCS/SCTS/BCIS also recommended administrative and management involvement. They also recommend that patients’ and caregivers’ input should be considered. In Europe, the EUROSCORE or EUROSCORE II is used for surgical risk calculation. Interestingly, there was a strong recommendation that centers without surgical capability should invite cardiac surgeons and interventional cardiologists with experience in complex PCI to participate via teleconference in an open format to promote transparency and to ensure discussion of all available treatment options^[9]^. It should be noted that there are several differences between European and American guidelines for the evaluation and management of patients with coronary artery disease, and these may impact how patients are ultimately treated^[36,37]^.

## WHICH PATIENTS SHOULD BE PRESENTED TO THE HEART TEAM

It remains unclear which patients should be discussed by the heart team. One approach in the UK and similarly in the Netherlands was to hold daily meetings and discuss all patients with coronary artery disease potentially requiring intervention^[38,39]^. Another approach is to focus on patients with complex coronary anatomy. An early study by Sanchez *et al*., defined complex coronary artery disease (CAD) in patients with one of the following: (1) unprotected left main CAD; (2) three-vessel CAD; (3) proximal single vessel LAD in patients with diabetes mellitus; and (4) any other cases where the treating physician felt that revascularization could reasonably be approached with either percutaneous or surgical strategies^[4]^. The authors concluded that whereas most patients met appropriate use criteria, the heart team approach can be used to account for both angiographic and clinical criteria in a multidisciplinary setting. In general, patients with significant left main coronary artery disease, complex coronary anatomy, intermediate or high SYNTAX scores, chronic total occlusions, multivessel disease, and disease at major bifurcations should be considered for discussion. The 2021 ACC/AHA/SCAI Guideline states that a Heart Team approach is recommended for patients for whom the optimal treatment strategy is unclear [Table 1]. Furthermore, more recent data has shown that medical management may be equivalent to an invasive strategy in stable ischemic coronary disease^[40]^. The heart team may be able to help delineate which patients will benefit from optimal medical management *versus* an intervention.

Additional reasons to discuss patients may include factors not captured with risk scores. Although risk score calculations such as the SYNTAX, Society of Thoracic Surgery - Predicted Risk of Mortality (STS-PROM), and EUROSCORE I and II contribute to the analysis of certain interventions, they do not encompass all of the clinical factors that may impact the potential outcome of an intervention. The SYNTAX II 2020 score is now available with the addition of clinical parameters in addition to coronary anatomy. As recommended in the 2021 ACC/AHA/SCAI guidelines, a heart team should consider these clinical variables that may impact the outcomes of revascularizaiton^[1]^. There may also be unique variables to consider, especially if they will affect the technical conduct of intervention. For example, a heart team discussion may be useful if a potential surgical patient has a porcelain aorta, a hostile chest, a lack of conduit, or anticipated difficult targets for grafting. Some comorbidities that may affect decision making include uncontrolled diabetes, severely decreased systolic function, frailty, advanced liver disease, advanced cancer, and other conditions [Table 1]. Furthermore, patients, primary care providers, and referring cardiologists should have the ability to request a multidisciplinary discussion as the situation dictates.

## ATTRIBUTES OF A SUCCESSFUL HEART TEAM

**Figure 1.**
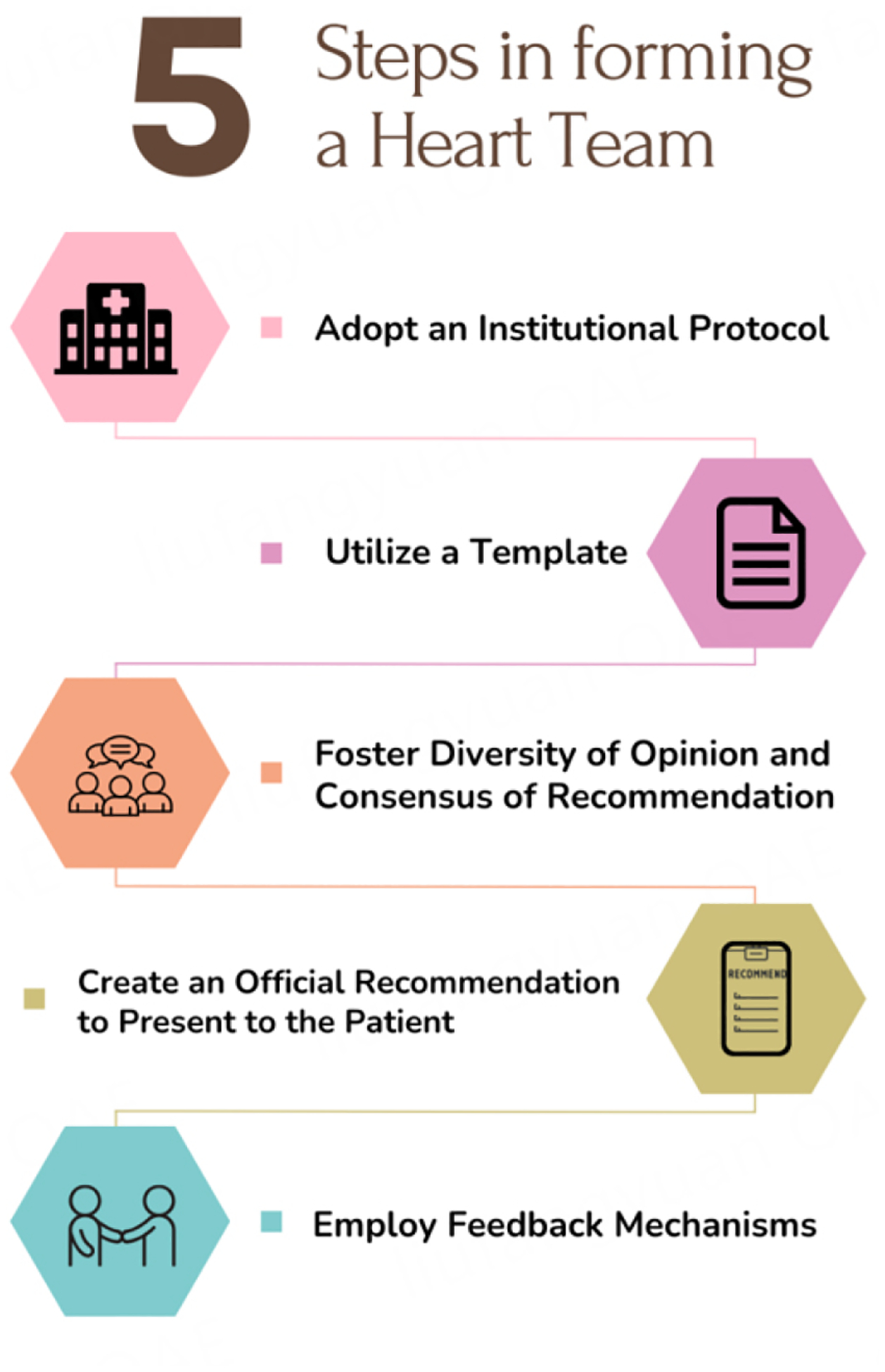
The “Five Star” model, adapted from Lee *et al*., is depicted here and has five steps to operationalizing a heart team^[41]^. These steps are intended for broad use to guide the formation of an effective heart team.

The multidisciplinary team model has been used in specialties other than cardiovascular medicine, including transplant medicine, oncology, critical care, and others. Within cardiovascular care, multidisciplinary teams have been used in congenital heart surgery, structural heart, advanced heart failure, cardiogenic shock, and others. One proposal for operationalizing a heart team is to use the “Five Star” model^[41]^. Teams would (1) adopt an institutional protocol; (2) utilize a template and scoring system; (3) foster diversity of opinion and consensus of recommendations; (4) create an official recommendation to present to the patient; and (5) employ feedback mechanisms [Figure 1]. These feedback mechanisms include feedback to the patients and referring providers about the heart team recommendations, feedback to the committee on overall guideline adherence and appropriate use criteria adherence, and long-term feedback on overall outcomes. Using these and similar approaches, other multidisciplinary teams have demonstrated decreased in-hospital costs, improved guideline adherence, improved interprofessional communication, and reproducibility of consistency in decision making^[27,41]^.

**Figure 2.**
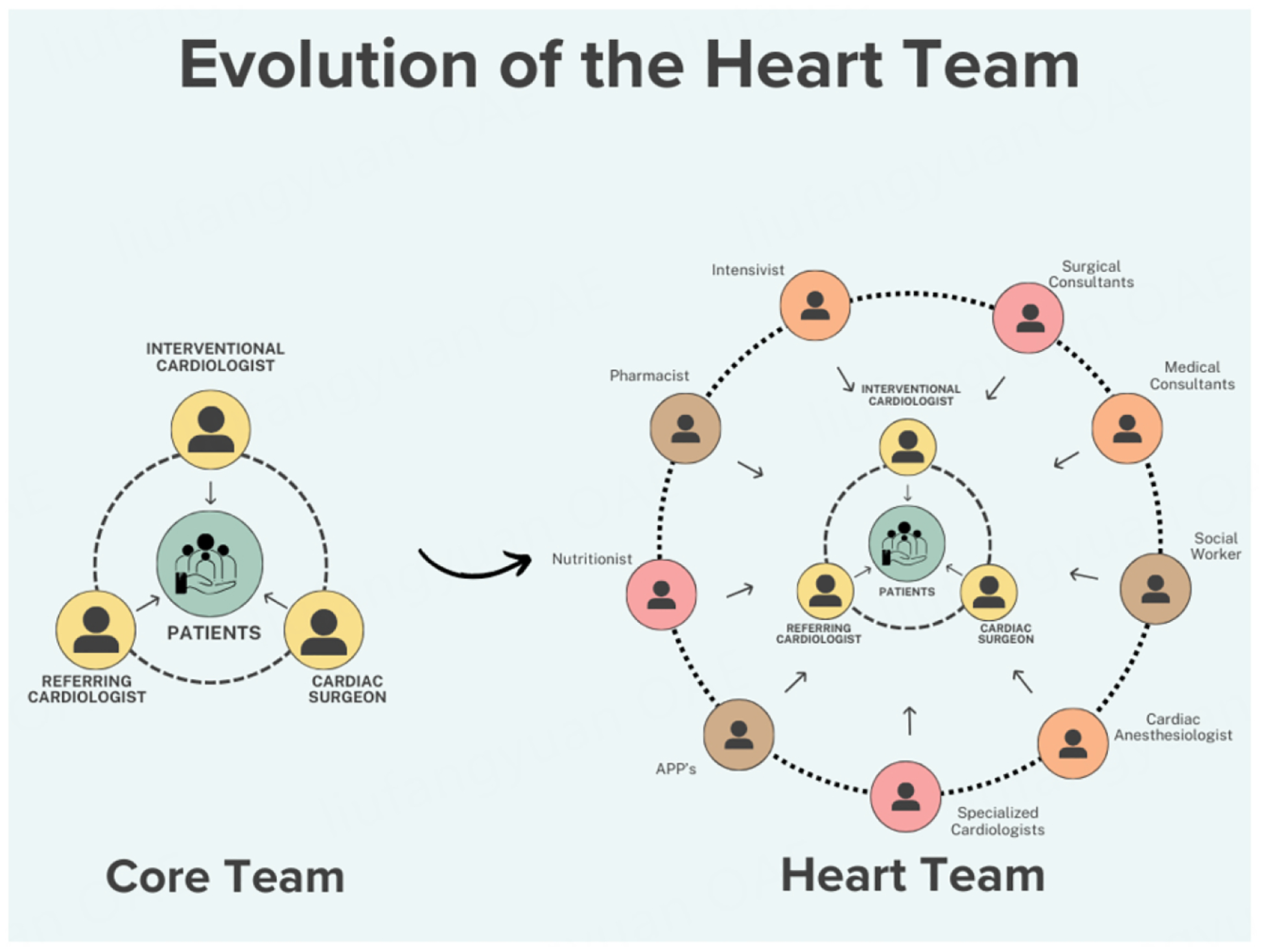
Evolution of the Heart Team: the heart team has evolved from a core group of members, which typically included an interventional cardiologist, cardiac surgeon, and the referring cardiologist or other physician. Current multidisciplinary heart teams may include additional medical and surgical consultants, specialized cardiologists, such as heart failure and echocardiography, intensivists, advanced practice providers (APP’s), clinical pharmacists, nutritionists, social workers, and others as needed to provide a more holistic, patient-centered approach to the decision-making process.

As mentioned previously, the core heart team should include a cardiologist, a cardiac surgeon, and an intensivist. Other members of the heart team may include primary cardiologists as well as interventional cardiologists, cardiac anesthesiologists, referring providers, hospitalists, other specialists, and advanced practice providers. Additional members may include ancillary staff such as clinical pharmacists, nutritionists, social workers, and others as needed [Figure 2]^[29]^. Typically, patients are presented in a structured format and pertinent imaging is reviewed^[19]^.

As with any project, there are several attributes that will lead to a successful heart team. The most successful teams will develop enduring goals. Such goals and objectives should be achievable, realistic, and measurable. A core team representative from cardiology and surgery should review and update the goals periodically. A review of the heart team decisions and outcomes is imperative as part of a quality improvement (QI) initiative. The QI initiative can be sponsored and evaluated by specific personnel trained in these measurements. This initiative should follow a continuum, and as new evidence and guidelines are published, the goals may need to be adjusted. A vital part of the heart team is uninhibited open communication among the major stakeholders. There should be strong leadership and mutual respect between team members. The atmosphere should maintain inclusivity and promote diversity and equity. It is also paramount that there is full transparency of the outcomes. If there is concern about the conduct of the team, goals, decisions, or outcomes, team members should feel at liberty to voice these concerns and a timely response should be made by the core members. The team should strive to engage in continuing education, employ new guidelines, and explore new treatment options. Of utmost importance, there should be a goal of patient-centered decision making^[35,42]^. This may be challenging and care should be taken to maintain HIPAA compliance. There should be an adequate facility or room with appropriate audiovisual equipment and teleconferencing capabilities. Finally, there should be financial and administrative support from the medical institution to promote the heart team. This may also include public verbal support, providing appropriate expectations to participate, and respecting the time commitments of stakeholders^[5,9,17]^.

## BARRIERS TO A SUCCESSFUL HEART TEAM

Although all providers would agree to any measures that would improve patient care and outcomes, there may be some barriers to the successful implementation of a heart team. One of the biggest hindrances may be the culture at the institution. Some physicians at larger institutions may be burdened with multiple meetings. There may be a lack of resources or space to hold a meeting. The various physicians may be at different locations. By creating buy-in from multiple stakeholders, these cultural hurdles can be overcome. While most heart team meetings had been face-to-face, one group showed that an online format was well attended and very efficient, with a 98.7% completion rate of referrals^[43]^. During the COVID-19 pandemic, many conferences resorted to using teleconference media^[44]^. Although the online format alleviated some of the barriers to holding a meeting, the large increase in the number of virtual meetings also created a sense of overwhelming time spent in teleconferences^[45]^. This “Zoom fatigue” has been associated with anxiety, feelings of social isolation, and emotional exhaustion^[46]^. To mitigate some of these effects, the heart team should aim to respect timely meetings, and equally reaffirm the value of efficient work and sociality^[47]^.

There may also be less participation in large online meetings *versus* face-to-face meetings, and institutions with limited technology support may have difficulty arranging online meetings^[44]^. The solution may be a hybrid approach where participants have the option of online *versus* in-person meetings.

Previously, one barrier to presenting patients may have been discussing patients of physicians not part of the primary institution, such as a primary cardiologist from a private practice group. Should the referring physicians from outside hospitals present the patients? How is the final recommendation of the heart team relayed back to the referring physician? Some of these communication barriers have been alleviated in recent years with the use of web-based platforms^[43]^, where a link can be sent to the outside referring physicians. If they do not participate, then one of the heart team members should inform the referring physician of the multidisciplinary recommendation, while taking the patient’s preference into consideration^[39]^.

One of the unique challenges in cardiovascular medicine is creating a balance between formulating a treatment plan and treating a patient in a timely fashion^[18]^. For example, a clinician may decide to perform PCI immediately after a diagnostic angiogram. Recently, a retrospective review of patients in the state of New York from 2017 to 2019 found that *ad hoc* PCI was still performed at a high rate, including 78% of patients with multivessel or left main coronary artery disease, 76% of patients with 3-vessel disease, and 75% of patients with diabetes^[14]^. Some of the arguments for *ad hoc* PCI may be a shorter time to revascularization, non-availability of cardiac surgery, patient preference, or other reasons. However, it may be beneficial to seek the viewpoints and input of a multidisciplinary team in the setting of complex coronary artery disease^[18]^. The effect is a delay in treatment. In fact, one group found an average delay of 7 days to PCI when a heart team was used^[48]^. Although they did not evaluate whether this delay led to adverse outcomes during the waiting period, they surmised that, based on other studies, delay in treatment could lead to increased major events prior to revascularization. The group from Erasmus University Medical Center in the Netherlands reported their utilization of the Heart team^[39]^. They held a daily meeting for 30 min to discuss all patients with CAD, including patients from their own institutions and from community hospitals. The median interval to review a patient from the time of referral was 2 days. The median interval to treatment was 12 days. Thus, not only did they demonstrate feasibility, but the safety of the heart team. In terms of timing, some hurdles may be difficult to avoid, such as when holidays or other important meetings supercede that of the heart team. There should be another mechanism for discussing patients in these situations, such as direct phone calls arranged to include the primary cardiologist, the interventional cardiologist, and the cardiac surgeon. The goal should be to identify which patients might have a survival advantage with a different treatment option, whether it is PCI, optimal medical therapy, or CABG surgery, and which can be achieved through a heart team consultation and shared decision making^[14]^.

Another major barrier is how to get the same benefit of the multidisciplinary discussion in an acute setting, such as a patient in cardiogenic shock, or on the weekends. One option is to create an emergent conference call or email chain where representatives from cardiology, surgery and the intensive care unit can discuss these patients and make a decision expeditiously. In tertiary care centers, a cardiogenic shock team may respond to these patients. Similar to the heart team, the shock team will have providers from ICU, interventional cardiology, cardiac surgery, and advanced cardiomyopathy services. The shock team can discuss immediate treatment options, resuscitation goals, advanced therapies, and revascularization options as indicated^[7]^. Similarly, a rapid response, multidisciplinary team to treat patients with pulmonary emboli has been developed at Vanderbilt^[8]^ and Massachusetts General Hospital^[49]^. A 24-h hotline is used to activate the pulmonary embolism response team (PERT), which includes specialists from emergency medicine, interventional cardiology, cardiac surgery, vascular surgery, pulmonology and critical care, and hematology. These teams for acute patients allowed expedited decision making based on approved algorithms and protocols.

Another concern is whether all relevant patients are discussed in the heart team conference. It may be difficult to determine whether all relevant patients are actually captured within such multidisciplinary conferences. For example, are there patients with clinical equipoise who preferentially undergo certain treatments at certain institutions? One group presented every patient with a new diagnosis of CAD in a heart team meeting^[50]^. Approximately 65% of patients were discussed *ad hoc*, and of those, 63% went on to PCI. On the other hand, 35% were discussed in the weekly Heart team meeting, and of these, approximately 87% went on to have surgery. Although this “all-comer” approach may ensure equity in patient selection, it can also be a large commitment of resources. Another approach may be to only present patients deemed high risk for PCI^[29]^. This approach reportedly had good participation of not only cardiologists and cardiac surgeons, but also other specialists whose involvement was not required. This limited approach to patient selection may have greater physician participation, but may inadvertently exclude patients with clinical equipoise, and raises the concern that maybe more patients should be presented in the heart team meetings^[51]^. Thus, the best approach may be to identify those patients who could be equally treated with either PCI or CABG, which was the approach of Patterson *et al*.^[52]^. However, even in this study, one limitation per the authors was that the determination of clinical equipoise was subjective and dependent on the referring physician.

As with many conferences, time is a major limiting factor for busy clinicians. There are also limitations to finding the most convenient time for multiple clinicians. There is also time required for preparation, and time that could potentially be used for clinical, educational, or research purposes^[17]^. Creating consistency will lead to participants prioritizing attendance at the meetings. There should be respect for everyone’s time, and discussions should be focused on pertinent issues. Some may view the time as a lost chance to generate clinical revenue or complete other clinical tasks. However, one study of multidisciplinary teams showed that multidisciplinary team evaluation of breast cancer patients resulted in an overall cost reduction to patient care^[53]^. Medical record documentation in the patient chart as a consult or progress note generates reimbursement and may encourage participation for some. This can be facilitated by the involvement of administrative staff in the multidisciplinary meetings to review coding practices and to ensure accuracy and optimize billing^[42]^.

## THE HEART TEAM OF JOHNS HOPKINS MEDICINE

Johns Hopkins Hospital is a major academic health center that performs a high volume of diagnostic cardiac catheterizations, percutaneous interventions, and cardiac surgery procedures yearly. At Johns Hopkins Hospital, there are two workflows to activate the Heart team. The most common pathway is the weekly Heart team discussion, which focuses on elective outpatient referrals. However, the Shock (acute heart) team activation via phone may be used when a decision is needed within 6–12 h and is for acutely ill inpatients, most of whom are in one of the intensive care units. Previously, elective meetings were held in person in a conference room, but now, the meetings are held via a web interface with HIPAA compliance. Multiple cardiology, interventional cardiology, intensivists, and cardiac surgery attendings are in attendance, as well as training fellows from general cardiology, interventional cardiology, cardiology critical care unit, and cardiac surgery. There is a minimum of one senior and often additional other junior attendings of these core specialties. Often, there are representatives from advanced practice providers from the step-down ward and the Cardiovascular Surgical intensive care unit. There may be additional providers from other specialties as warranted, such as ethics, infectious disease, radiology or other imaging experts, pulmonary medicine, oncology, psychiatry, *etc*. There is often a list of patients to be discussed, or providers may spontaneously present a new patient at their discretion. Although not required, a few slides are used to present background information, and then there is a succinct presentation of relevant imaging, including recent angiograms, echocardiograms, computed tomography scans, and cardiac MRI if available. The discussion focuses on the main issues of debate or concern, with references to expert consensus or current guidelines. The STS-PROM, SYNTAX II score, or other predictive scoring calculators are presented as needed. Finally, the Heart team strives to achieve a consensus decision on the optimal treatment plan or additional information or testing as needed. The referring provider will document this plan and supporting information in the patient’s chart. Another unique facet of the Heart team conference is that providers may subsequently provide follow-up information, including outcomes of the interventions performed. On a routine basis, members will present patients who have undergone the recommended intervention and the overall outcome. Here, we present examples of complex patients who were presented at our weekly multidisciplinary heart team discussion.

### Patient 1

A 58-year-old man with obesity and gastroesophageal reflux disease presented with angina and elevated troponin and was transferred from another hospital. On coronary angiogram, he had multivessel CAD that was not amenable to PCI; echocardiogram showed reduced biventricular function with left ventricular ejection fraction (LVEF) of 15%−20%, and a cardiac MRI showed mostly viable myocardium. A CT chest also demonstrated an ascending aortic aneurysm of 5 cm. This patient was presented to the Heart Team to discuss options for treatment or intervention, centered around the discussion of whether he should go for CABG *versus* heart transplant or durable left ventricular assist device (LVAD) implantation. He was deemed not a good candidate for PCI. There were multiple suggestions, including medical management, off-pump CABG, pump-assist CABG, on-pump CABG with temporary LVAD assist, and complete heart transplant/LVAD workup prior to CABG in the event he is on prolonged mechanical support. Per Heart Team recommendations and per 2021 ACC/AHA/SCAI guidelines for coronary revascularization^[1]^ based on severe left main disease, he successfully underwent an on-pump one-vessel CABG, LIMA to LAD, as the other vessels did not appear graftable. He also had an ascending aortic aneurysm replacement, and placement of a temporary LVAD into the aorta for support after cardiopulmonary bypass.

### Patient 2

**Figure 3.**
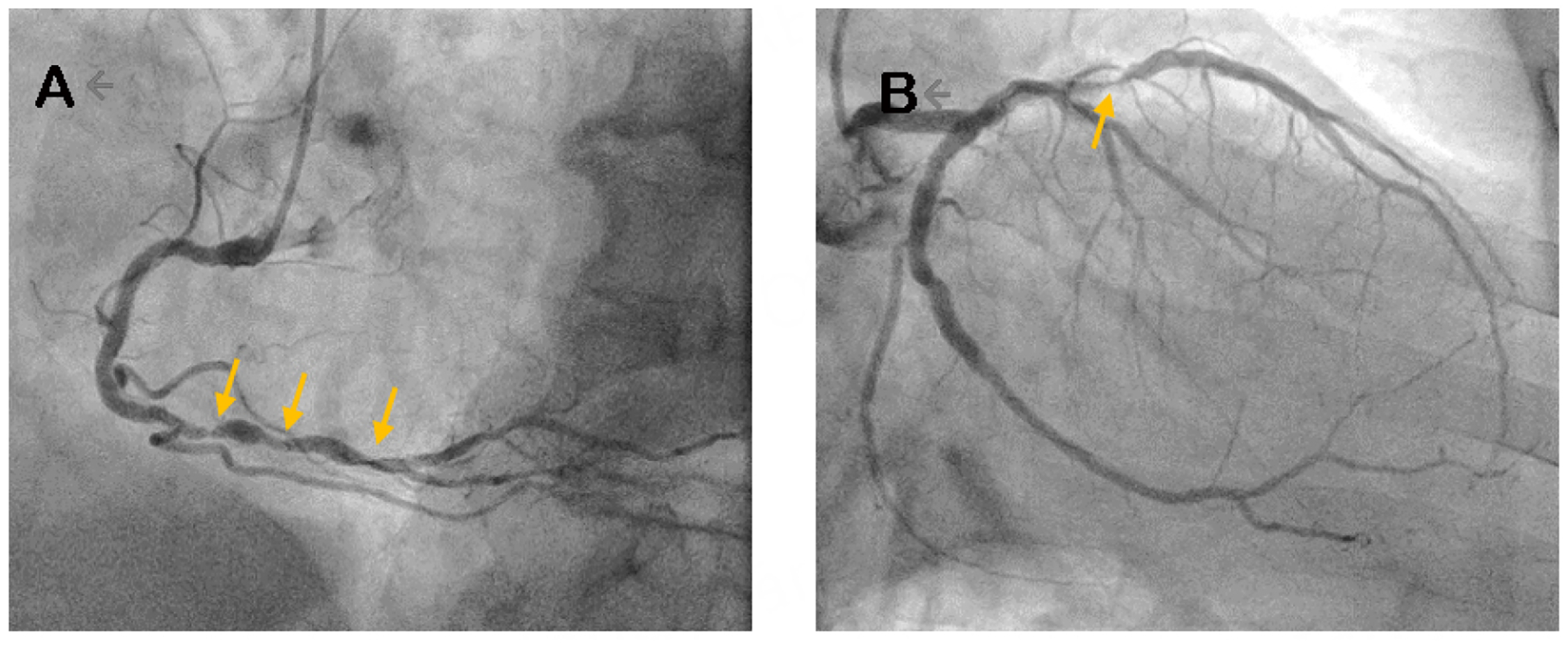
Coronary angiogram images of a 75-year-old man with a history of STEMI and PCI to RCA, LAD, and LCF who presented with angina and was discussed by a Heart Team (Patient #2). (A) representative image of the right coronary artery showing several areas of distal stenoses (yellow arrows). (B) A representative image of the left coronary arteries and in-stent restenosis of the LAD (yellow arrow). STEMI: ST elevation myocardial infarction; PCI: percutaneous intervention; RCA: right coronary artery; LAD: left anterior descending; LCF: left circumflex.

A 75-year-old man with complex coronary artery disease, status post PCI to the RCA 13 years prior and PCI to the left anterior descending and left circumflex in the setting of a STEMI 6 years prior, was presented to the Heart Team. Additional past medical history included hypertension, obesity with a BMI of 38.9 kg/m^2^, current smoker, and recent gastrointestinal bleeding after starting apixaban for thrombosis of the greater saphenous vein. He presented with angina and further workup demonstrated an LVEF 40%−45%, multivessel CAD, including in-stent restenosis of the proximal to mid LAD [Figure 3]. He had no acceptable saphenous vein due to prior surgeries, and stenosis of the right subclavian artery. In this case, the patient was presented for Heart Team discussion as he was seen as a high-risk patient due to obesity, limited conduit, and current tobacco use. Stenosis of the proximal right subclavian artery precluded the use of the *in-situ* RIMA, and there was concern that a free RIMA would not reach from the aorta to the PDA. There was a discussion about optimal medical management *versus* intervention. One option was to use a free RIMA as a T graft off the LIMA, but several surgeons agreed this was too risky for possible injury to the LIMA. On the other hand, the PCI option was suboptimal as this would have required multiple overlapping stents from the ostium to the mid LAD. After discussion with the Heart team, there was consensus that the best option would be a hybrid approach and to proceed with a one-vessel CABG using the LIMA to the LAD. This was in agreement with the 2021 ACC/AHA/SCAI Guideline for coronary artery revascularization; the use of the LIMA as a conduit to bypass the LAD is a class 1 recommendation. He underwent successful surgery and was discharged home. A plan was to continue optimal medical management and PCI to the RCA only if he subsequently developed angina.

### Patient 3

**Figure 4.**
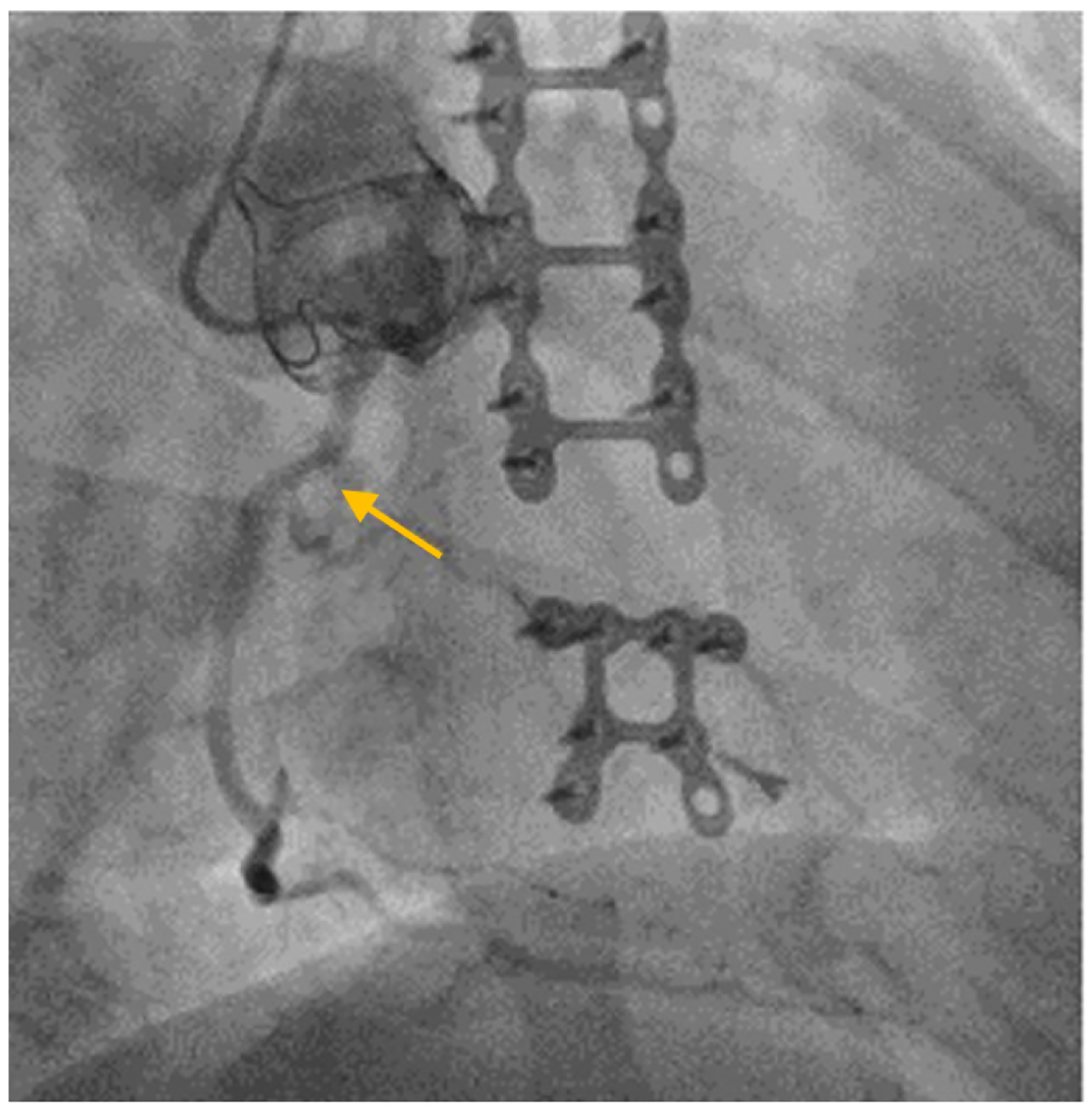
Coronary angiogram of a 71-year-old male with a history of AVR and MVr complicated by sternal wound infection and closure with titanium plates and screws and pectoralis muscle flaps who presented with severe AS, severe MS, and CAD and was discussed by a Heart Team (Patient #3). A representative image demonstrates significant stenosis in the proximal RCA (yellow arrow), contrast in the ascending aorta, sternal plates and screws, and bioprosthetic aortic valve. AVR: Aortic valve replacement; MVr: mitral valve repair; AS: aortic stenosis; MS: mitral stenosis; CAD: coronary artery disease; RCA: right coronary artery.

A 71-year-old man with a history of bicuspid aortic valve status post aortic valve replacement with a 25 mm CE Perimount Magna bioprosthetic valve and mitral valve repair with a 34 mm Cosgrove band annuloplasty about 10 years prior. Post-operatively, he underwent sternal wound debridement and eventual sternal fixation with plates and bilateral pectoralis flap advancement. That hospital course was also significant for heparin-induced thrombocytopenia. On workup, he was found to have moderate pulmonary hypertension, reduced cardiac index, severe bioprosthetic aortic valve stenosis, moderate to severe mitral stenosis, and severe RCA stenosis [Figure 4]. The surgeon recommended a re-do sternotomy, aortic valve replacement, mitral valve replacement and a one-vessel CABG to the RCA, but the patient was a high-risk surgical candidate. He wanted to explore other options, so he was presented at the Heart Team conference. During the interim, he was placed on optimal medical therapy. The Heart team discussion included his primary cardiologist, interventional cardiologists, structural heart cardiologists, and surgeons. Although the patient could potentially undergo percutaneous coronary intervention and transcatheter aortic valve-in-valve, the structural heart cardiologists recommended against a transcatheter approach to his mitral valve since he had an incomplete mitral annulus band, which was not ideal for a transcatheter approach due to the anatomic constraints given the need for concomitant aortic valve-in-valve. Thus, the recommendation of the Heart Team was to proceed with surgery based on the 2020 ACC/AHA Guideline for the management of patients with valvular heart disease, which has a Class 2 recommendation for CABG if a patient is undergoing a concomitant valvular surgery^[54]^. The patient underwent high-risk re-do sternotomy, aortic valve replacement, mitral valve replacement, and one-vessel CABG. This case also highlights the patientcenteredness and shared-decision making, which is also a Class 1 recommendation^[1]^. He had an acceptable postoperative course and is recovering well.

## OTHER EXAMPLES OF HEART TEAM CONFERENCES

At Johns Hopkins, there are several other heart teams including a structural heart team, a heart transplant team, an extracorporeal membrane oxygen/cardiogenic shock (ECMO) team, and a pulmonary embolus response team (PERT). The structural heart team and the heart transplant team follow a very structured weekly meeting where every patient being considered for intervention is reviewed prior to surgery or medical management. The ECMO and PERT teams serve to address acutely decompensating patients. Providers contact the central call center, which then emergently contacts multiple providers on one call. Team members include a cardiologist, intensivist, surgeon, and other interventionalists as needed. The referring provider gives a quick patient synopsis and review of current data. Then, the team decides on the best course of action while on one call.

At the University of Pittsburgh Medical Center, PA, a daily heart team meeting discusses referred patients with unprotected left main CAD, multivessel CAD, proximal LAD disease in diabetic patients or other patients with complex CAD that could potentially benefit from either PCI or CAD. The patient clinical presentation includes a brief summary of the clinical presentation, comorbid conditions, calculated SYNTAX and STS scores, measurement of left ventricular function, and subjective commentary on the patients’ overall functional status and frailty. The information is collected on a data sheet, and the Heart team discusses different approaches and optimal treatment strategies^[18]^. Notably, this team met multiple times per week to discuss patients. The authors noted increased collaboration between cardiologists and surgeons, and an unmeasurable educational benefit for those involved in the meetings.

Similarly, the Massachusetts General Hospital (MGH) established a Heart Team as part of a quality improvement program^[29]^. The MGH Heart Team evaluated patients with complex CAD and other high-risk features such as comorbidities or socioeconomic factors. Most notably, the MGH Heart Team generated a unique and succinct structured form that contained a summary of pertinent information such as medical history, laboratory data, angiography and other imaging information, risk scores, and a summary to mark what the recommended treatment is based on most recent guidelines. This highly structured format undoubtedly facilitated succinct presentations, as well as a means to track outcomes. The MGH Heart Team noted that most of the patients were older, had more complex comorbidities, and had higher SYNTAX and STS risk scores. Among all PCI and CABG patients, there was a low in-hospital mortality rate of 3.9%, a low observed-to-expected ratio of 30-day mortality in the CABG group, and better adherence to guideline recommendations for PCI, suggesting improved outcomes after Heart Team discussion based on most current evidence^[29]^.

## FUTURE CONSIDERATIONS

The ultimate goal of the heart team is to provide high-quality, patient-centered care based on established guidelines and a detailed review of the individual patient’s social and medical situation with input from the patient, cardiologists, surgeons, and other specialists as needed. The multidisciplinary team should have commitment from the providers and also full support from the hospital administration. This may be in the form of accepting the time put forth as clinical time, providing the infrastructure and administrative support to run an effective heart team, and recognizing the efforts of team members. In the future, institutions may consider mandates or requirements for the establishment of a heart team, but this should maintain a collegiate and collaborative atmosphere. Currently, the preprocedural assessment and Heart Team approach is a recommended structural measure in 2023 AHA/ACC Clinical Performance and Quality Measures for Coronary Artery Revascularization^[55]^. Once a Heart Team has been established, a goal for improvement would be to expand the access to the heart team via telemedicine to smaller branch hospitals, including clinics and hospitals not a part of the main institution. This will not only benefit referral patterns but, more importantly, will allow for more equitable inclusion of patients in the respective communities. In larger metropolitan areas, this could lead to competition between major institutions. However, as long as there is mutual respect, the competition will benefit the community and improve patient outcomes. Another potential benefit of a telemedicine platform is that nationally and internationally renowned providers may be able to discuss some of the more challenging scenarios. Again, this will create a collaborative atmosphere and bear further credence to the heart team.

Finally, the rapid growth of artificial intelligence (AI) will eventually play an important role in the evaluation of coronary artery disease^[56]^ and the evaluation by a heart team. At the time of writing this manuscript, there was no specific example of AI use in heart team decision making. AI is based on using machine learning algorithms. These algorithms are like a set of instructions for computer programs on how to analyze data. Data are defined using a structured description, called an ontology. Machine learning algorithms use an ontology to interpret and understand data from various sources^[57]^. One benefit of machine learning algorithms is that they can be used to process a massive amount of unstructured data in an unbiased format^[58]^. A machine learning classification model could be utilized to predict the impact of an intervention based on the patient’s complex comorbidities and procedural risks. Then, a machine learning regression model could be utilized to predict the outcomes after an intervention. A unique feature of machine learning models is that they will continue to evolve as more data are gathered. Specific machine learning algorithms could potentially be developed for each unique institution, and treatment and evaluation recommendations would be based on major guidelines and reflective of the best outcomes within that hospital system. Finally, a programming interface would allow a physician to interact with the model and ask questions in a variety of contexts to optimize the outcome^[58]^. With the plethora and ever-increasing amounts of data, AI may play a vital role in future Heart Team discussions.

As the population survival increases, there will be new challenges, potentially new causes of disease, and new treatment modalities, and the heart team will be the center of guiding decision making in complex coronary artery disease.

## Figures and Tables

**Table 1.** Factors considered by the Heart Team, adapted from the 2021 ACC/AHA/SCAI Guidelines^[1]^

Comorbidities
Diabetes
Systolic dysfunction
Coagulopathy
Valvular heart disease
Frailty
Malignant neoplasm
End-stage renal disease
Chronic obstructive pulmonary disease
Immunosuppression
Debilitating neurological disorders
Liver disease/cirrhosis
Prior CVA
Calcified/porcelain aorta
Aortic aneurysm
**Procedural factors**
Local and regional outcomes
Access site for PCI
Surgical risk
PCI risk
**Patient factors**
Unstable presentation or shock
Patient preferences
Inability or unwillingness to adhere to DAPT
Patient social support
Religious beliefs
Patient education, knowledge, and understanding
